# The Triple-Decker Complex [Cp*Fe(µ,η^5^:η^5^-P_5_)Mo(CO)_3_] as a Building Block in Coordination Chemistry

**DOI:** 10.3390/molecules24020325

**Published:** 2019-01-17

**Authors:** Mehdi Elsayed Moussa, Stefan Welsch, Luis Dütsch, Martin Piesch, Stephan Reichl, Michael Seidl, Manfred Scheer

**Affiliations:** Department of Inorganic Chemistry, University of Regensburg, 93040 Regensburg, Germany; Mehdi.Elsayed-Mousa@chemie.uni-regensburg.de (M.E.M.); stefan-welsch@gmx.de (S.W.); Luis.duetsch@chemie.uni-regensburg.de (L.D.); Martin.piesch@chemie.uni-regensburg.de (M.P.); Stephan.reichl@chemie.uni-regensburg.de (S.R.); michael1.seidl@chemie.uni-regensburg.de (M.S.)

**Keywords:** triple-decker, *cyclo*-P_5_, weakly coordinating, molybdenum, silver, copper

## Abstract

Although the triple-decker complex [Cp*Fe(µ,η^5^:η^5^-P_5_)Mo(CO)_3_] (**2**) was first reported 26 years ago, its reactivity has not yet been explored. Herein, we report a new high-yielding synthesis of **2** and the isolation of its new polymorph (**2’**). In addition, we study its reactivity towards Ag^I^ and Cu^I^ ions. The reaction of **2** with Ag[BF_4_] selectively produces the coordination compound [Ag{Cp*Fe(µ,η^5^:η^5^-P_5_)Mo(CO)_3_}_2_][BF_4_] (**3**). Its reaction with Ag[TEF] and Cu[TEF] ([TEF]^−^ = [Al{OC(CF_3_)_3_}_4_]^−^) leads to the selective formation of the complexes [Ag{Cp*Fe(µ,η^5^:η^5^-P_5_)Mo(CO)_3_}_2_][TEF] (**4**) and [Cu{Cp*Fe(µ,η^5^:η^5^-P_5_)Mo(CO)_3_}_2_][TEF] (**5**), respectively. The X-ray structures of compounds **3**–**5** each show an M^I^ ion (M^I^ = Ag^I^, Cu^I^) bridged by two P atoms from two triple-decker complexes (**2**). Additionally, four short M^I^···CO distances (two to each triple-decker complex **2**) participate in stabilizing the coordination sphere of the M^I^ ion. Evidently, the X-ray structure for compound **3** shows a weak interaction of the Ag^I^ ion with one fluorine atom of the counterion [BF_4_]^−^. Such an Ag···F interaction does not exist for compound **4**. These findings demonstrate the possibility of using triple-decker complex **2** as a ligand in coordination chemistry and opening a new perspective in the field of supramolecular chemistry of transition metal compounds with phosphorus-rich complexes.

## 1. Introduction

For more than two decades, the chemistry of transition metal complexes bearing polyphosphorus ligands has been an active area of research for both inorganic and organometallic chemists [1]. The interest in this class of compounds does not only originate from the large variety of bonding patterns in the formed products, but is also due to their potential as building blocks in supramolecular chemistry [1,2]. Until now, the most successful candidates in this field have been polyphosphorus (P_n_) complexes, such as the Cp* derivative of pentaphosphaferrocene [Cp*Fe(η^5^-P_5_)] (**1**, Cp* = η^5^-C_5_Me_5_) [3]. Its reaction with Ag^I^, Cu^I^ and group 13 metal salts (Tl^I^, In^I^, Ga^I^) of the weakly coordinating anion [Al{OC(CF_3_)_3_}_4_]^−^ ([TEF]^−^) allows the formation of one-dimensional polymers with various coordination modes of the P_5_ unit [4,5,6,7,8]. With Cu^I^ halides and **1**, depending on the reaction conditions, a set of one- and two-dimensional coordination polymers [9,10], fascinating fullerene-like spherical aggregates [11,12,13,14,15,16] as well as an organometallic nanosized capsule [17] are accessible. Moreover, when ditopic organic linkers are added to **1** and Cu^I^ halides, unprecedented organometallic–organic hybrid polymers with various dimensionalities can be realized [18,19].

Besides its exciting supramolecular chemistry, the *cyclo*-P_5_ complex **1** was used as a suitable starting compound for the synthesis of a number of triple-decker complexes with *cyclo*-P_5_ middle-decks. Based on **1**, the Scherer group prepared the 30-electron triple-decker complexes [CpFe(µ,η^5^:η^5^-P_5_)FeCp*]^+3^ and [Cp*Fe(µ,η^5^:η^5^-P_5_)M(CO)_3_] (M = Cr, Mo) [20,21]. Kudinov et al. reported a few iron-, ruthenium- and molybdenum-containing triple-decker complexes such as [Cp*Fe(µ,η^5^:η^5^-P_5_)MCp*]^+^ (M = Fe, Ru) and [(C_7_H_7_)Mo(µ,η^5^:η^5^-P_5_)FeCp*]^+^ [22,23,24]. More recently, our group used **1** to synthesize several unique neutral triple-decker sandwich complexes with functionalized *cyclo*-P_5_ middle-decks [25]. In addition to these examples, some others continuously appear in the literature with *cyclo*-P_5_ middle-decks synthesized from starting materials other than **1** [26,27,28].

Regardless of the ongoing progress in the synthesis of this class of triple-decker compounds, their reactivity and especially their coordination chemistry are totally unexplored. In fact, to the best of our knowledge, apart from the few coordination compounds of the [(Cp*Mo)_2_(µ,η^6^:η^6^-P_6_)] triple-decker complex recently reported by our group [29], no coordination chemistry of any other triple-deck with any *cyclo*-P_n_ middle-deck has been reported as yet. Accordingly, we studied the potential of triple-decker complexes with *cyclo*-P_5_ middle-decks as multidentate ligands in supramolecular chemistry. The goal of such studies is to understand the coordination chemistry of these complexes with transition metal salts and compare it to that of the complex [Cp*Fe(η^5^-P_5_)] (**1**). Herein, we present a new high-yielding synthesis of the triple-decker complex [Cp*Fe(µ,η^5^:η^5^-P_5_)Mo(CO)_3_] (**2**), and we show that its reaction with the coinage metal salts Ag[BF_4_], Ag[TEF] and Cu[TEF] allows the formation of the three unprecedented coordination compounds: [Ag{Cp*Fe(µ,η^5^:η^5^-P_5_)Mo(CO)_3_}_2_][BF_4_] (**3**), [Ag{Cp*Fe(µ,η^5^:η^5^-P_5_)Mo(CO)_3_}_2_][TEF] (**4**) and [Cu{Cp*Fe(µ,η^5^:η^5^-P_5_)Mo(CO)_3_}_2_][TEF] (**5**), respectively. 

## 2. Results and Discussion

### 2.1. Synthesis and X-Ray Structure of [Cp*Fe(µ,η^5^:η^5^-P_5_)Mo(CO)_3_] *(**2**)*

**Scheme 1:**
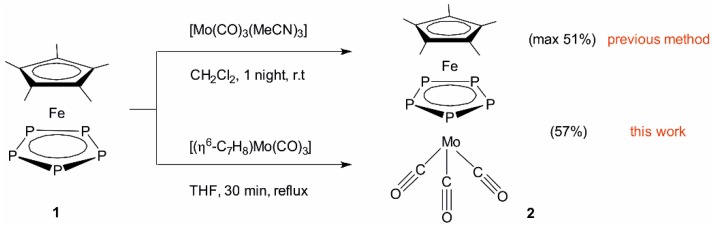
Previous and current methods used for the synthesis of triple-decker complex **2**.

The previously reported synthesis of the triple-decker complex **2** from the *cyclo*-P_5_ complex **1** and [Mo(CO)_3_(CH_3_CN)_4_] requires an extended chromatographic purification. Moreover, it is only partially reproducible, as several attempts isolated **2** with very different yields (10–40%) that were all lower than the initially reported one (51%) [21], which is probably one reason why the chemistry of this compound has not been studied despite it having been discovered almost three decades ago. Therefore, we developed another method for the synthesis of **2**, involving the 1:1 reaction of the *cyclo*-P_5_ complex **1** with [(η^6^-C_7_H_8_)Mo(CO)_3_] in THF instead of [Mo(CO)_3_(CH_3_CN)_4_] in CH_2_Cl_2_ (Scheme 1). Although this new method requires a higher temperature than the one previously reported, it only takes 30 minutes instead of one night and has a higher yield (57%) and better reproducibility (for further details, see Section 3.2.1). In addition, chromatographic purification can be avoided by removing the THF solvent and recrystallizing the product from the crude mixture using the appropriate amount of CH_2_Cl_2_. Interestingly, this procedure additionally allowed the crystallization of a new polymorph, **2’**, of the triple-decker complex **2**.

**Figure 1:**
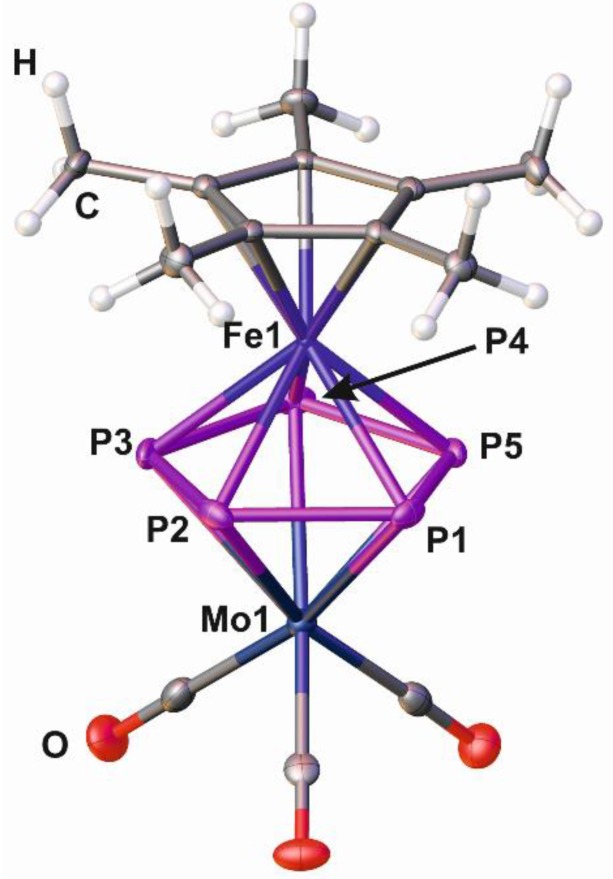
Molecular structure of **2’** in the solid state. Selected bond lengths [Å] and angles [°]: Fe1–P1 2.3822(8), Fe1–P2 2.3812(8), Fe1–P3 2.4010(8), Fe1–P4 2.3665(8), Fe1–P5 2.4097(7), Mo1–P1 2.6548(8), Mo1–P2 2.6579(7), Mo1–P3 2.5843(8), Mo1–P4 2.7036(7), Mo1–P5 2.5694(7), P1–P2 2.1611(1), P2–P3 2.1453(1), P3–P4 2.1582(1), P4–P5 2.1561(1), P5–P1 2.1475(1), Mo1–P1-Fe1 85.59(2), Mo1–P2–Fe1 85.53(2), Mo1–P3–Fe1 86.79(2), Mo1–P4–Fe1 84.80(2), Mo1–P5–Fe1 86.95(2).

Compound **2’** crystallizes in the space group *C*2/*c* of the orthorhombic crystal system (**2**; monoclinic *P*2_1_/*n*). The geometry of **2’** is mostly similar to that of **2** (Figure 1). The P–P bond lengths in **2’** are slightly longer (2.1453(1)–2.1611(1) Å) than those in **2** (2.116(6)–2.138(8) Å). The average Fe–P distance is also slightly longer in **2’** (2.388(1) Å) than in **2** (2.370(4) Å). The mean Mo–P distances are identical within the margin of error (2.634(1) Å, 2.630(4) Å). The *cyclo*-P_5_ ring in **2’** is approximately planar as in **2** (maximum deviation: 0.064(1) Å). Due to the slightly longer P–P distances, a slightly shorter intermetallic distance between Fe and Mo is found for **2’** (3.428 Å) than for **2** (3.443 Å). The new structure determination of **2’** was carried out at a lower temperature (101 K) than that of **2** (r.t.) and showed better quality factors and a lower residual electron density. For this reason, the metric data of **2’** are used in this paper for geometric comparisons.

### 2.2. Synthesis and X-Ray Structure of the Coordination Compound [Ag{Cp*Fe(µ,η^5^:η^5^-P_5_)Mo(CO)_3_}_2_][BF_4_] *(**3**)*

**Scheme 2:**
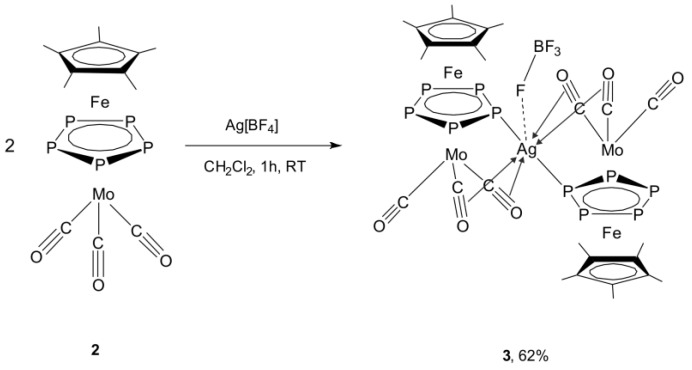
The reaction of triple-decker complex **2** with Ag[BF_4_]. Synthesis of the coordination compound **3**.

As a first approach, the triple-decker complex **2** was reacted with Ag[BF_4_]. This reaction was carried out in CH_2_Cl_2_ at room temperature with a 2:1 stoichiometric ratio and led to the selective isolation of complex [Ag{Cp*Fe(µ,η^5^:η^5^-P_5_)Mo(CO)_3_}_2_][BF_4_] (**3**) in a good (62%) yield (Scheme 2). 

**Figure 2:**
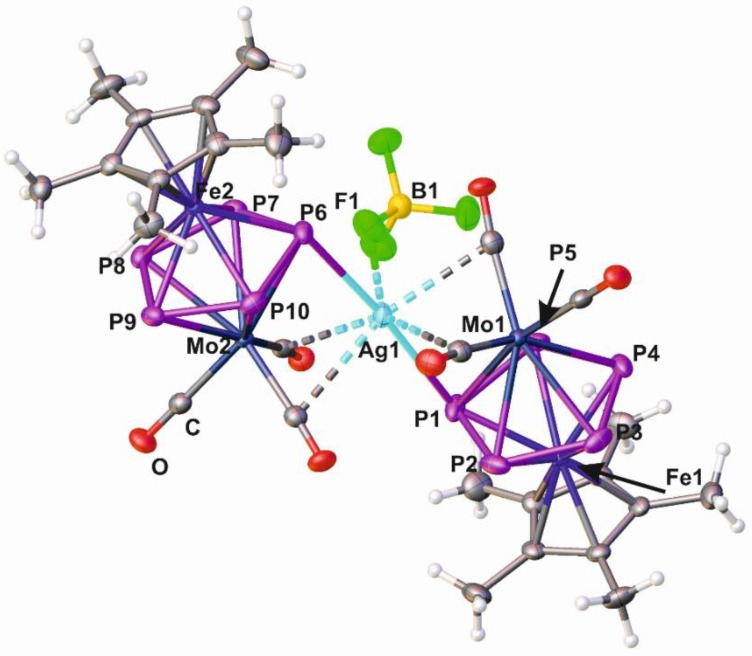
Molecular structure of **3** in the solid state. Selected bond lengths [Å] and angles [°]: Ag1–P1 2.7683(1), Ag1–P6 2.7149(1), Ag1–F1 2.622(3), P1–P2 2.1670(2), P2–P3 2.1465(2), P3–P4 2.1668(2), P4–P5 2.1368(2), P5–P1 2.1660(2), P6–P7 2.1711(1), P7–P8 2.1429(1), P8–P9 2.1721(1), P9–P10 2.1531(1), P10–P6 2.1745(1), P1–Ag1–P6 175.09(4), P1–Ag1–F1 99.07(7), P6–Ag1–F1 83.45(6), Mo1–Ag1–Mo2 155.93(3).

Dark red crystals of **3**, which are suitable for single crystal X-ray diffraction, were obtained after layering the crude reaction mixture with *n*-pentane (Figure 2). Compound **3** crystallizes in the monoclinic space group *P*2_1_/*c*. The asymmetric unit contains one Ag^I^ cation, two complexes of **2,** and one BF_4_^−^ anion. Two P atoms coordinate to the Ag^I^ ion, one from each of the two complexes **2** (Figure 2), with Ag–P distances of 2.7149(1) Å and 2.7683(1) Å, respectively. Additionally, four short distances to the C atoms of the two CO ligands of both complexes **2** were identified ($\bar{d}$(Ag···CO) = 2.726 Å). A possible bonding mode for these CO ligands makes them bind to one Mo atom in a common terminal fashion and also donate electron density from the CO π bonds to the Ag atom. This bonding pattern of CO ligands has only been rarely reported in the literature [30]. Moreover, the Ag1–F1 distance is short (2.622(3) Å) and falls within the limits of van der Waals interactions (2.30–2.90 Å) [31]. Overall, the Ag^I^ cation shows a distorted pentagonal bipyramidal coordination geometry. The average P–P bond length in **3** is slightly longer than that in the free complex **2’** (2.160 Å compared to 2.154(1) Å; Table 1). The Mo–C distances of the semi-bridging CO ligands [32] are slightly longer than the ones of the terminal CO ligands (mean values: 2.008 Å and 1.981 Å). The C–O bond lengths range between 1.136(5) and 1.147(5) Å. The Mo–C–O angles of the semi-bridging CO ligands deviate more from a linear geometry (173.6(3)–176.3(3)°) than those of the terminal ones (179.0(4)° and 179.2(3)°). The CO bands in the IR spectra of **3** are comparable to those of the free ligand. Therefore, it is supposed that the interaction between the cation Ag^⁺^ and the bridging CO ligands is weak. The Ag···Mo distances are relatively short at 2.8301(4) Å and 2.8442(4) Å. The room temperature ^1^H and ^13^C{^1^H} NMR spectra of **3** in CD_2_Cl_2_ show characteristic signals for the Cp* and CO ligands. In the ^31^P{^1^H} spectrum, in CD_2_Cl_2_, a broad singlet at 25.2 ppm can be detected, which is significantly shifted to lower field compared to the free ligand (9.7 ppm). The signal for the [BF_4_]^−^ anion in the ^19^F{^1^H} NMR spectrum in CD_2_Cl_2_ at −151.7 ppm is broadened due to the interaction with the Ag^+^ cation (ω_1/2_ = 84 Hz).

### 2.3. Synthesis and X-Ray Structure of the Coordination Compound [Ag{Cp*Fe(µ,η^5^:η^5^-P_5_)Mo(CO)_3_}_2_][TEF] *(**4**)*

**Scheme 3:**
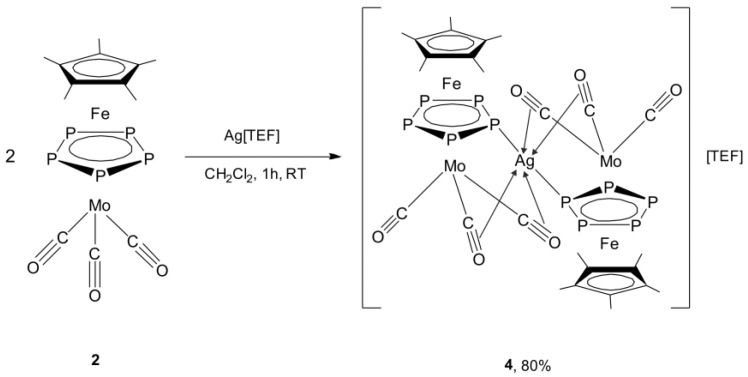
The reaction of the triple-decker complex **2** with Ag[TEF]. Synthesis of complex **4**.

The result obtained from the reaction of complex **2** with Ag[BF_4_] raised the question of the possibility of expanding this chemistry to other Ag^I^ salts. Accordingly, we selected the Ag^I^ salt of the weakly coordinating anion [TEF]^−^ because it offers a high solubility, thus allowing a more detailed study of the formed compounds in solution [33]. The reaction of **2** with Ag[TEF], performed by applying reaction conditions similar to those used for the reaction with Ag[BF_4_], afforded the selective isolation of the complex [Ag{Cp*Fe(µ,η^5^:η^5^-P_5_)Mo(CO)_3_}_2_][TEF] (**4**) with an excellent yield (80%, Scheme 3).

**Figure 3:**
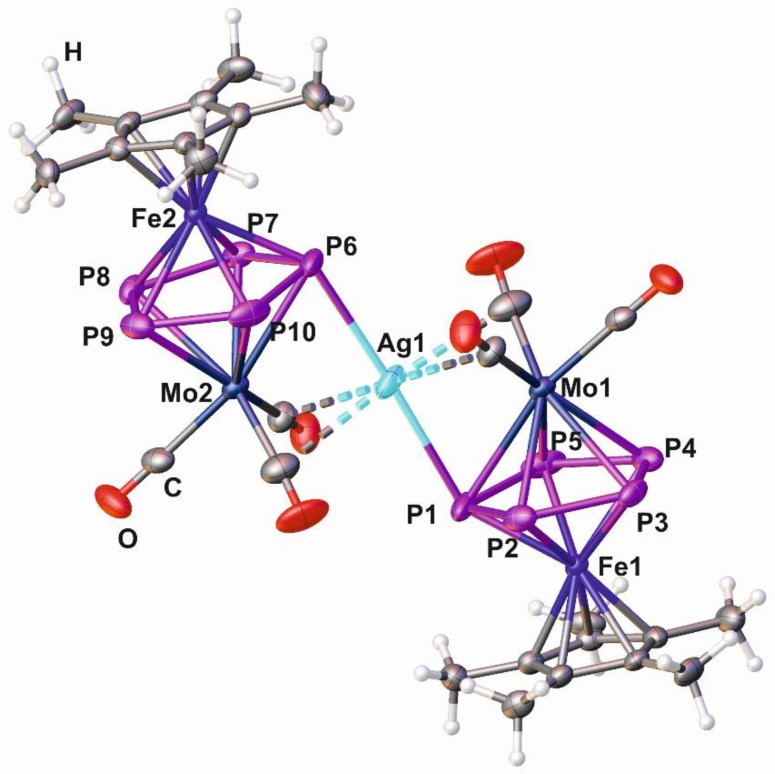
Molecular structure of the cation of **4** in the solid state. Selected bond lengths [Å] and angles [°]: Ag1–P1 2.7097(2), Ag1–P6 2.6809(2), P1–P2 2.173(3), P2–P3 2.139(3), P3–P4 2.159(3), P4–P5 2.147(3), P5–P1 2.168(3), P6–P7 2.166(3), P7–P8 2.137(3), P8–P9 2.164(3), P9–P10 2.159(3), P10–P6 2.169(3), P1–Ag1–P6 177.12(7), Mo1–Ag1–Mo2 173.56(3).

Single crystals of **4** were obtained by layering the CH_2_Cl_2_ crude reaction mixture with *n*-pentane (Figure 3). Compound **4** crystallizes in the monoclinic space group *P*2_1_/*c*. Similar to what was observed for **3**, the crystal structure of **4** shows an Ag^I^ cation coordinated by two P atoms with Ag–P distances of 2.6809(2) Å and 2.7097(2) Å. Additionally, it exhibits four weak interactions with the C atoms of four CO ligands ($\bar{d}$(Ag···CO) = 2.748 Å). In contrast to **3**, no additional interactions are observed between the Ag^I^ cation and any fluorine atom from the [TEF]^−^ ion, probably due to the non-coordinating property of the [TEF]^−^ anion. As a consequence, the Ag^I^ ion adopts a distorted square bipyramidal coordination sphere in contrast to the pentagonal bipyramidal one observed for the Ag^I^ ion in **3**. Interestingly, the Mo–Ag–Mo angle in **4** (173.56(3)°) deviates much less from linearity than that in **3** (155.93(3)°), and the Ag···Mo distances in **4** (2.8131(7) Å and 2.8353(7) Å) are slightly shorter than those found in **3**.

As expected, compound **4** is excellently soluble in CH_2_Cl_2_ due to its [TEF]^−^ anion, whereas it is moderately soluble in toluene and insoluble in *n*-alkanes. The ^1^H, ^13^C{^1^H}, and ^19^F NMR spectra of **4** in CD_2_Cl_2_ at r.t. show characteristic signals for the Cp* and CO ligands as well as for the [TEF]^−^ anion. In the ^31^P{^1^H} NMR spectrum, a singlet at 30.1 ppm is observed, which is significantly shifted to lower field compared to the free ligand (9.7 ppm). Upon cooling to 183 K, no broadening or significant shift of the signal occur. The ^31^P{^1^H} MAS-NMR spectrum of solid **4** at room temperature shows a singlet at a chemical shift (28.3 ppm) similar to that in solution. Thus, molecular dynamic processes such as the rotation of the *cyclo*-P_5_ ring take place at low temperatures in solution and in the solid state at room temperature as well. However, the single-crystal X-ray analysis performed at 100 K did not indicate a rotation of the *cyclo*-P_5_ ring because of the low temperature needed for an appropriate structure solution. The molecular ion peak can be detected in the cation mode of the ESI mass spectrum as well as peaks for the intact and partially decarbonylated ligands of **2**. In the anion mode, the [TEF]^−^ anion is detected. Vapor pressure osmometry yields a relatively high value for the mean molecular mass (1700 g∙mol^−1^ ± 100 g∙mol^−1^), suggesting that the compound is mainly associated in solution. The CO stretching frequencies in the IR spectrum in the solid state are broadened compared to the one of the free ligand and are shifted to higher wave numbers, probably due to the positive charge of the complex (Table 1). In the ^13^C{^1^H} MAS-NMR spectrum, the signals for the C atoms of the carbonyl groups are broadened as well and, interestingly, a differentiation between semi-bridging and non-bridging CO ligands is not possible.

### 2.4. Synthesis and X-Ray Structure of the Complex [Cu{Cp*Fe(µ,η^5^:η^5^-P_5_)Mo(CO)_3_}_2_][TEF] *(**5**)*

**Scheme 4:**
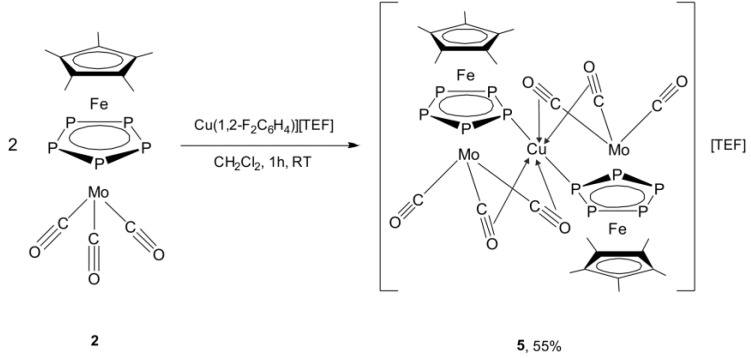
The reaction of triple-decker complex **2** with Cu(1,2-F_2_C_6_H_4_)[TEF]. Synthesis of the coordination compound **5**.

The successful coordination of triple-decker complex **2** to Ag^I^ ions inspired us to study its coordination behavior towards Cu^I^ ions, specifically towards [Cu(1,2-F_2_C_6_H_4_)][TEF]. This reaction was performed under similar reaction conditions as previously used and selectively yielded the complex [Cu{Cp*Fe(µ,η^5^:η^5^-P_5_)Mo(CO)_3_}_2_][TEF] (**5**) in good isolated yields (55%, Scheme 4).

**Figure 4:**
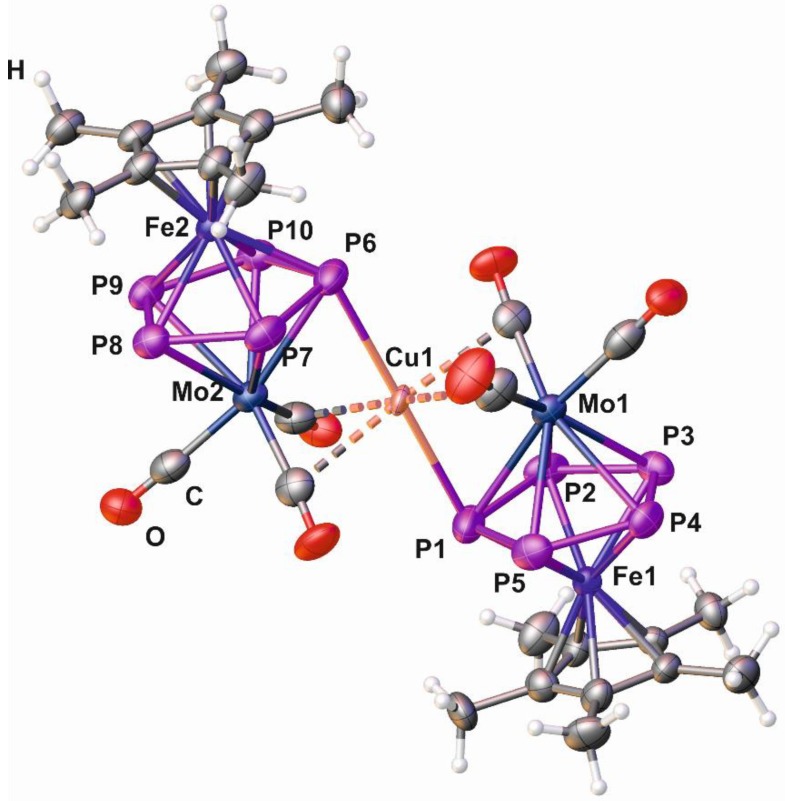
Molecular structure of the cation of **5** in the solid state.

Compound **5** crystallizes in the monoclinic space group *P*2_1_/*c*. The asymmetric unit contains two independent complexes [Cu(**2**)_2_]^+^ and two [TEF]^−^ anions, which show slightly different bonding lengths and angles (entitled in the following as **5’** and **5’’**). The monocationic units in **5** (**5’**, **5’’**) exhibit the same geometry and coordination modes as the related Ag^I^ compound **4** (Figure 4). The Cu–P bond distances in **5** lie between 2.647(2) Å and 2.691(2) Å and the short contacts between Cu and the semi-bridging CO ligands range from 2.534 Å to 2.645 Å. Table 1 shows a comparison of selected structural and spectroscopic data of the independent molecules of **5’** and **5’’** as well as of the silver-containing compounds **3** und **4** with the same ligand. The average P–P distances in the silver compounds are slightly larger than in the copper compound, but they are all elongated compared to the free triple-decker complex **2**’. The Cu–P distances are relatively large, but smaller than the Ag–P distances, as expected. The P–M–P angle amounts to nearly 180° in all cases. The largest deviation from linearity is observed in the [BF_4_]^−^ compound **3** with about 5°. The intermetallic distances within the copper compound are significantly smaller than those in the silver compounds. The complexes with [TEF]¯ as counterion show Mo–M–Mo angles between 173.56(3)° and 174.92(4)°, while compound **3** clearly differs from these (155.93(3)°).

In the ^31^P{^1^H} NMR spectra, all compounds show a low field shift compared to the signal of free complex **2**. The difference in the chemical shift is larger for [TEF]^−^ than for [BF_4_]^−^ and larger for copper than for silver. The CO bands in the IR spectra of **3** are almost similar to those of the starting material. However, in regard to compounds **4** and **5**, they are broadened and shifted to higher wave numbers. In the ESI mass spectra of **5**, the molecular ion peak can be detected in the cation mode, as in the case of **4**, as well as peaks for the intact and partially decarbonylated ligands. In the anion mode, only a single peak can be observed for the [TEF]^−^ anion. 

## 3. Materials and Methods 

### 3.1. General Information

All experiments were performed under an atmosphere of dry argon using standard Schlenk techniques. [(C_7_H_8_)Mo(CO)_3_] and Ag[BF_4_] were purchased from Sigma-Aldrich (Darmstadt, Germany) and used as received without further purification. The compound [Cp*Fe(η^5^-P_5_)] (**1**) [3] and the salts Ag[Al{OC(CF_3_)_3_}_4_] [33] and Cu(1,2-F_2_C_6_H_4_)[Al{OC(CF_3_)_3_}_4_] [34] were synthesized according to literature procedures. Solvents were freshly distilled under argon from Na/benzophenone (THF), from Na (toluene), from CaH_2_ (CH_2_Cl_2_) and from a Na/K alloy (*n*-pentane, *n*-hexane). IR spectra were recorded on a Varian FTS-800 spectrometer (Varian Inc., MA, USA). ^1^H, ^13^C, ^31^P and ^19^F NMR spectra were recorded on Bruker Avance 300 and 400 spectrometers (Bruker, Karlsruhe, Germany). MAS-NMR spectra were performed at 300 K on a Bruker Avance 300 spectrometer (Bruker, Karlsruhe, Germany). ^1^H and ^13^C NMR chemical shifts were reported in parts per million (ppm) relative to Me_4_Si as an external standard. ^31^P NMR chemical shifts were expressed in ppm relative to external 85% H_3_PO_4_ and were decoupled from the proton. ^19^F NMR chemical shifts were reported relative to CFCl_3_. For the ESI-MS, a Finnigan Thermoquest TSQ 7000 mass spectrometer (Thermo Fisher Scientific, Frankfurt, Germany) was used. Elemental analyses were performed by the microanalytical laboratory of the University of Regensburg. Molecular mass determination performed by means of vapor pressure osmometry was carried out using a Knauer K-7000 vapor pressure osmometer (Knauer, Berlin, Germany) with CH_2_Cl_2_ as the solvent.

### 3.2. Synthesis and Characterization of the Compounds ***2*** and ***3***–***5***

#### 3.2.1. Synthesis and Characterization of [Cp*Fe(µ,η^5^:η^5^-P_5_)Mo(CO)_3_] (**2**)

To a stirred solution of [Cp*Fe(η^5^-P_5_)] (**1**, 1.28 g, 3.69 mmol) in 75 mL THF, [(C_7_H_8_)Mo(CO)_3_] (1 g, 3.69 mmol) in 75 mL THF was slowly added. The crude reaction mixture was refluxed for 30 minutes. The solvent was removed under reduced pressure and the residue was extracted with 50 mL CH_2_Cl_2_. In order to obtain crystals of the polymorph **2**’, the crude mixture was stored for three days at −30 °C. Otherwise, the crude residue mixture was purified by column chromatography using silica gel (10 × 2 cm) and an eluent solvent mixture of hexane:toluene (9:1). The triple-decker complex **2** was obtained as a dark green solution, the solvent was removed under reduced pressure and **2** was isolated as an olive-green solid. Yield: (1.11 g, 57%). Crystal Data for C_13_H_15_O_3_P_5_FeMo, *M_r_* = 525.89 g/mol, monoclinic, *C*2/*c* (No. 15), a = 15.8694(11) Å, b = 14.6679(10) Å, c = 15.9594(12) Å, *β* = 102.587(7)°, *α* = *γ* = 90°, *V* = 3625.6(5) Å^3^, *T* = 101(2) K, *Z* = 8, *Z′* = 1, *μ*(CuK*_a_*) = 16.348, 10281 reflections measured, 3185 unique (*R_int_* = 0.0235), which were used in all calculations. The final *wR_2_* was 0.0699 (all data) and the *R_1_* was 0.0237 (I > 2(I)).

#### 3.2.2. Synthesis and Characterization of [Ag{Cp*Fe(µ,η^5^:η^5^-P_5_)Mo(CO)_3_}_2_][BF_4_] (**3**)

A solution of Ag [BF_4_] (18 mg, 0.09 mmol) and [Cp*Fe(µ,η^5^:η^5^-P_5_)Mo(CO)_3_] (**2**; 95 mg, 0.18 mmol) in CH_2_Cl_2_ (15 mL) was stirred at r.t. for 1 h in the dark. Subsequently, the deep red solution was filtered through a G4 filter frit and layered with *n*-pentane (25 mL) using a Teflon capillary. Within two weeks, red crystals of **3** had formed after storage at 2 °C. These were filtered off, washed with *n*-pentane (3 × 2 mL), and dried in a vacuum. Yield (69 mg, 62%)

^1^H NMR (300 MHz, CD_2_Cl_2_): *δ* = 1.36 ppm (s, C_5_(CH_3_)_5_), ^13^C{^1^H} NMR (75.47 MHz, CD_2_Cl_2_): *δ* = 9.8 (br sext, ^3^*J*_P,C_ = 1.8 Hz; C_5_(CH_3_)_5_), 89.0 (br s, C_5_(CH_3_)_5_), 214.3 ppm (br s, CO). ^31^P{^1^H} NMR (121.49 MHz, CD_2_Cl_2_): *δ* = 25.2 ppm (s, ω_1/2_ = 28 Hz). ^19^F{^1^H} NMR (282.40 MHz, CD_2_Cl_2_): *δ* = −151.7 ppm (br s, ω_1/2_ = 84 Hz; CF_3_). IR (KBr): ῦ/cm^−1^ = 2957 (vw), 2906 (w), 2852 (vw), 1963 (vs), 1955 (vs), 1892 (vs), 1880 (vs), 1477 (w), 1447 (vw), 1427 (w), 1376 (w), 1309 (w) 1242 (m), 1209 (w), 1155 (w), 1124 (w), 1071 (w), 1020 (m), 986 (w), 600 (w), 584 (w), 557 (w), 544 (w) 493 (m), 477 (m), 440 (w). IR (CH_2_Cl_2_): ῦ/cm^−^^1^ = 3054 (vs), 2987 (w), 2349 (vw), 2305 (vw), 1970 (vs), 1892 (vs), 1897 (vs), 1422 (m), 1267 (vs). Elemental analysis, calcd. (%) for C_26_H_30_AgBF_4_Fe_2_Mo_2_O_6_P_10_ (1246.46 g·mol^−1^): C 25.05, H 2.43; found: C 25.81, H 2.54. Crystal Data for C_104_H_120_Ag_4_B_4_F_16_Fe_8_Mo_8_O_24_P_40_, *M_r_* = 4985.83 g·mol^−1^, monoclinic, *P*2_1_/*c* (No. 14), a = 8.77020(10) Å, b = 26.5995(3) Å, c = 19.5513(3) Å, *β* = 116.2050(10)°, *α* = *γ* = 90°, *V* = 4092.20(10) Å^3^, *T* = 123(2) K, *Z* = 1, *Z′* = 0.25, *μ*(CuK*_a_*) = 18.417, 16649 reflections measured, 7769 unique (*R_int_* = 0.0232), which were used in all calculations. The final *wR_2_* was 0.0955 (all data) and *R_1_* was 0.0354 (I > 2(I)).

#### 3.2.3. Synthesis and Characterization of [Ag{Cp*Fe(µ,η^5^:η^5^-P_5_)Mo(CO)_3_}_2_][TEF] (**4**)

A solution of [Ag(CH_2_Cl_2_)][TEF] (104 mg, 0.09 mmol) and two equivalents of [Cp*Fe(µ,η^5^:η^5^-P_5_)Mo(CO)_3_] (**2**; 95 mg, 0.18 mmol) in CH_2_Cl_2_ (15 mL) was stirred at r.t. for 1 h in the dark. The deep red solution was then filtered through a G4 filter frit and layered with *n*-pentane (25 mL) using a Teflon capillary. Within two weeks, red crystals of **4** had formed at 2 °C. These were filtered off, washed with *n*-pentane (3 × 2 mL), and dried in a vacuum. The mother liquor was further concentrated to 5 mL under vacuum, under which more product precipitated as a reddish brown precipitate upon addition of *n*-pentane (45 mL), likewise filtered off, washed with *n*-pentane (3 × 2 mL), and dried under vacuum. Yield (154 mg, 80%).

^1^H NMR (400 MHz, CD_2_Cl_2_): *δ* = 1.34 ppm (s, C_5_(CH_3_)_5_), ^13^C{^1^H} NMR (100.61 MHz, CD_2_Cl_2_): *δ* = 9.7 (br sext, ^3^*J*_P,C_ = 1.8 Hz; C_5_(CH_3_)_5_), 90.3 (s, C_5_(CH_3_)_5_), 121.6 (q, ^1^*J*_F,C_ = 292.1 Hz; CF_3_), 213.9 ppm (br sext, ^2^*J*_P,C_ = 1.6 Hz; CO). ^13^C{^1^H}-MAS-NMR (75.47 MHz): *δ* = 9.0 (br s, C_5_(CH_3_)_5_), 92.5 (br s, C_5_(CH_3_)_5_), 122.3 (q, ^1^*J*_F,C_ = 337.4 Hz; CF_3_), 215.1 ppm (br s, CO). ^31^P{^1^H} NMR (161.98 MHz, CD_2_Cl_2_): *δ* = 30.1 ppm (s, ω_1/2_ = 4 Hz). ^31^P{^1^H} NMR (161.98 MHz, CD_2_Cl_2_, 183 K): *δ* = 30.3 ppm (s, ω_1/2_ = 6 Hz). ^31^P{^1^H}-MAS-NMR (121.49 MHz): *δ* = 28.3 ppm (br s, ω_1/2_ = 93 Hz). ^19^F{^1^H} NMR (282.40 MHz, CD_2_Cl_2_): *δ* = −75.6 ppm (s, CF_3_). IR (KBr): ῦ/cm^−1^ = 2962 (vw), 2918 (w), 2851 (vw), 1980(vs), 1928 (vs), 1914 (vs), 1543 (vw), 1480 (w), 1450 (w), 1428 (w), 1381 (m), 1353 (m), 1301 (vs), 1277 (vs), 1242 (vs), 1219 (vs), 1165 (m), 1136 (vw), 1075 (vw), 1021 (m), 974 (vs), 833 (w), 799 (vw), 755 (vw), 727 (s), 587 (m), 559 (w), 538 (m), 492 (w), 476 (w), 444 (m). IR (CH_2_Cl_2_): ῦ/cm^−1^ = 2984 (vw), 2959 (vw), 2915 (vw), 2849 (vw), 1993(vs), 1970 (vs), 1934 (s), 1902 (vs), 1478 (w), 1448 (vw), 1426 (m), 1379 (m), 1352 (m), 1300 (vs), 1277 (vs), 1242 (vs), 1225 (vs), 1167 (m), 1136 (vw), 1072 (vw), 1022 (m), 976 (vs), 833 (vw), 600 (w), 587 (w), 559 (w), 538 (w), 494 (w), 476 (vw), 444 (m). Osmometry (CH_2_Cl_2_): Average molar mass: 1700 g·mol^−1^ ± 100 g·mol^−1^. Positive ion ESI-MS (CH_2_Cl_2_), *m*/*z* (%) = 1159.0 (7) [Ag{Cp*FeP_5_Mo(CO)_3_}_2_]^+^, 527.9 (27) [Cp*FeP_5_Mo(CO)_3_]⁺, 499.9 (100) [Cp*FeP_5_Mo(CO)_2_]⁺. negative ion ESI-MS (CH_2_Cl_2_), *m*/*z* (%) = 967.2 (100) [Al{OC(CF_3_)_3_}_4_]^−^. Elemental analysis, calcd. (%) for C_42_H_30_AgAlF_36_Fe_2_Mo_2_O_10_P_10_ (2126.79 g·mol^−1^): C 23.72, H 1.42; found: C 23.73, H 1.44. Melting point > 200 °C. Crystal Data for C_42_H_30_AgAlF_36_Fe_2_Mo_2_O_10_P_10_, *M_r_* = 2126.79 g·mol^−1^, monoclinic, *P*2_1_/*c* (No. 14), a = 19.6554(16) Å, b = 9.6564(9) Å, c = 36.745(3) Å, *β* = 98.912(9)°, *α* = *γ* = 90°, *V* = 6889.9(10) Å^3^, *T* = 100(2) K, *Z* = 4, *Z′* = 1, *μ*(CuK*_a_*) = 12.144, 26789 reflections measured, 12207 unique (*R_int_* = 0.0444), which were used in all calculations. The final *wR_2_* was 0.1467 (all data) and *R_1_* was 0.0498 (I > 2(I)).

#### 3.2.4. Synthesis and Characterization of [Cu{Cp*Fe(µ,η^5^:η^5^-P_5_)Mo(CO)_3_}_2_][TEF] (**5**)

A solution of [Cu(1,2-F_2_C_6_H_4_)][TEF] (92 mg, 0.08 mmol) and [Cp*Fe(µ,η^5^:η^5^-P_5_)Mo(CO)_3_] (**2**; 84 mg, 0.16 mmol) in CH_2_Cl_2_ (15 mL) was stirred at r.t. for 1 h. Subsequently, the deep red solution was then filtered through a G4 filter frit and layered with *n*-pentane (25 mL) using a Teflon capillary. Within two weeks, red crystals of **5** had formed at 2 °C, which were filtered off, washed with *n*-pentane (3 × 2 mL), and dried in a vacuum (5 h, 10^−3^ mbar). According to the elemental analysis, half an equivalent of *n*-pentane per unit formula could not be removed (The asymmetric unit of **5** also contains half an *n*-pentane molecule). Yield (93 mg, 55%).

^1^H NMR (300 MHz, CD_2_Cl_2_): *δ* = 1.38 ppm (s, C_5_(CH_3_)_5_), ^13^C{^1^H} NMR (100.61 MHz, CD_2_Cl_2_): *δ* = 9.7 (br s; C_5_(CH_3_)_5_), 90.5 (s, C_5_(CH_3_)_5_), 121.6 (q, ^1^*J*_F,C_ = 291.4 Hz; CF_3_), 212.9 ppm (br s; CO). ^31^P{^1^H} NMR (121.49 MHz, CD_2_Cl_2_): *δ* = 31.7 ppm (s, ω_1/2_ = 41 Hz). ^19^F{^1^H} NMR (282.40 MHz, CD_2_Cl_2_): *δ* = −75.6 ppm (s, CF_3_). IR (KBr): ῦ/cm^−1^= 2962 (vw), 2919 (w), 2854 (vw), 1993(vs), 1961(vs), 1923 (vs), 1910 (vs), 1479 (w), 1451 (w), 1428 (w), 1381 (m), 1353 (m), 1302 (vs), 1277 (vs), 1241 (vs), 1220 (vs), 1166 (m), 1139 (vw), 1072 (vw), 1021 (m), 974 (vs), 833 (w), 756 (vw), 728 (vs), 586 (w), 561 (w), 537 (m), 492 (w), 475 (w), 443 (m). IR (CH_2_Cl_2_): ῦ/cm^−1^ = 3054 (vs), 2987 (w), 2348 (w), 2305 (vw), 1995 (s), 1969 (vs), 1925 (m), 1901 (s), 1422 (m), 1269 (vs). Positive ion ESI-MS (CH_2_Cl_2_), *m*/*z* (%) = 1114.6 (1) [Cu{Cp*FeP_5_Mo(CO)_3_}_2_]^+^, 527.9 (100) [Cp*FeP_5_Mo(CO)_3_]^+^, 499.9 (94) [Cp*FeP_5_Mo(CO)_2_]^+^. Negative ion ESI-MS (CH_2_Cl_2_), *m*/*z* (%) = 967.1 (100) [Al{OC(CF_3_)_3_}_4_]^−^. Elemental analysis, calcd. (%) for C_42_H_30_CuAlF_36_Fe_2_Mo_2_O_10_P_10_ × 0.5C_5_H_12_ (2118.55 g·mol^−1^): C 25.23, H 1.71; found: C 25.36, H 1.73. Crystal Data for C_86.5_H_66_Al_2_Cu_2_F_72_Fe_4_Mo_4_O_20_P_20_, *M_r_* = 4200.99, monoclinic, *P*2_1_/*c* (No. 14), a = 32.1751(3) Å, b = 12.2014(2) Å, c = 35.8672(5) Å, *b* = 99.6750(10)°, *a* = *g* = 90°, *V* = 13880.5(3) Å^3^, *T* = 123(2) K, *Z* = 4, *Z′* = 1, *m*(CuK*_a_*) = 10.176, 50354 reflections measured, 24416 unique (*R_int_* = 0.0606) which were used in all calculations. The final *wR_2_* was 0.1722 (all data) and *R_1_* was 0.0609 (I > 2(I)).

### 3.3. Crystallography

The crystals were selected and mounted on an Oxford Diffraction Gemini R Ultra diffractometer equipped with a Ruby CCD detector (**2**, **4**) and a SuperNova diffractometer equipped with an Atlas CCD detector (**3**, **5**), respectively. The crystals of compounds **2** and **4** were kept at T = 100(1) K and the crystals of the compounds **3** and **5** at T = 123(1) K during data collection, respectively. Data collection and reduction were performed with **CrysAlispro** (Version 171.33.41 (**2**), 171.34.9 (**3**), 171.33.42 (**4**), 171.33.61 (**5**)) [35]. For compounds **2** and **4**, a semi-empirical multi-scan absorption correction from equivalents [35] was applied. For compounds (**3**, **5**), an analytical numeric absorption correction using a multifaceted crystal model based on expressions derived by R. C. Clark & J. S. Reid was applied [36]. Using **Olex2** [37], the structures were solved with **SIR97** [38] and a least-square refinement on F2 was carried out with **ShelXL** [39]. All non-hydrogen atoms were refined anisotropically. Hydrogen atoms at the carbon atoms were located in idealized positions and refined isotropically according to the riding model. CIF files with comprehensive information on the details of the diffraction experiments and full tables of bond lengths and angles for **2**–**5** were deposited in Cambridge Crystallographic Data Centre under the deposition codes CCDC-1884710-1884713.

## 4. Conclusions

In conclusion, we found a new high-yielding synthesis of the triple-decker complex [Cp*Fe(µ,η^5^:η^5^-P_5_)Mo(CO)_3_] (**2**) and used it as a ligand in coordination chemistry for the first time. Its reaction with Ag[BF_4_], Ag[TEF] or Cu[TEF] leads, in each case, to the selective formation of complexes with the general formula [M{Cp*Fe(µ,η^5^:η^5^-P_5_)Mo(CO)_3_}_2_][X] (M = Ag, Cu; X = [BF_4_]^−^, [TEF]^−^). These results open a new chapter in the reactivity of triple-decker complexes with *cyclo*-P_5_ middle-decks as promising candidates in supramolecular chemistry. Current studies are focused on extending the new synthesis approach to obtain other triple-decker compounds with *cyclo*-P_5_ and *cyclo*-As_5_ middle-decks and studying their coordination chemistry towards transition metal complexes.

## Figures and Tables

**Table 1:** Comparison of structural and spectroscopic data of the compounds [M(**2**)_2_][X] and the triple-decker complex **2’**.

	2’	M = Ag [X] = [BF_4_] 3	M = Ag [X] = [TEF] 4	M = Cu [X] = [TEF] 5’	M = Cu [X] = [TEF] 5’’
$\bar{d}$(P,P)/Å	2.154(1)	2.160	2.158	2.156	2.154
$\bar{d}$(M,P)/Å	―	2.742	2.695	2.687	2.662
$\bar{d}$(Mo,M)/Å	―	2.837	2.824	2.680	2.673
∡(P,M,P)/°	―	175.09(3)	177.12(7)	179.61(9)	176.89(8)
∡(Mo,M,Mo)/°	―	155.93(3)	173.56(3)	174.92(4)	173.93(4)
*δ*^31^P{^1^H}/ppm CD_2_Cl_2_, RT	9.7	25.2	30.1	31.7	
ῦ_CO_/cm^-1^ KBr	1963, 1955, 1894, 1881	1963, 1955, 1892, 1880	1980 (br), 1928, 1914	1993, 1961, 1923, 1910	
